# Similarities and differences in the nuclear genome organization within Pooideae species revealed by comparative genomic in situ hybridization (GISH)

**DOI:** 10.1007/s13353-016-0369-y

**Published:** 2016-10-14

**Authors:** Joanna Majka, Maciej Majka, Michał Kwiatek, Halina Wiśniewska

**Affiliations:** Institute of Plant Genetics, Polish Academy of Sciences, Strzeszyńska 34, 60-479 Poznań, Poland

**Keywords:** Pooideae, *Brachypodium*, Cereals, Forage grasses, Comparative genomics, In situ hybridization

## Abstract

In this paper, we highlight the affinity between the genomes of key representatives of the Pooideae subfamily, revealed at the chromosomal level by genomic in situ hybridization (GISH). The analyses were conducted using labeled probes from each species to hybridize with chromosomes of every species used in this study based on a “round robin” rule. As a result, the whole chromosomes or chromosome regions were distinguished or variable types of signals were visualized to prove the different levels of the relationships between genomes used in this study. We observed the unexpected lack of signals in secondary constrictions of rye (RR) chromosomes probed by triticale (AABBRR) genomic DNA. We have also identified unlabeled chromosome regions, which point to species-specific sequences connected with disparate pathways of chromosome differentiation. Our results revealed a conservative character of coding sequence of 35S rDNA among selected species of the genera *Aegilops*, *Brachypodium*, *Festuca*, *Hordeum*, *Lolium*, *Secale*, and *Triticum*. In summary, we showed strong relationships in genomic DNA sequences between species which have been previously reported to be phylogenetically distant.

## Introduction

The Poaceae family (Barnh., Gramineae Juss.) is the fourth largest group of plants on Earth and can be found in nearly all regions and climate zones. More than 10,000 species and 600 genera constitute this family, but only three subfamilies, namely Ehrhartoideae, Pooideae, and Panicoideae, play the principal role of providing the human race with nutrition and a source of renewable energy. The Pooideae subfamily includes some economically important cereals, such as bread wheat (*Triticum aestivum* L.), barley (*Hordeum vulgare* L.), rye (*Secale cereale* L.), and triticale (×*Triticosecale* Wittm.), as well as many lawn and pasture grasses, such as perennial ryegrass (*Lolium perenne* L.). The genome composition of the species within Pooideae differs in respect to their size, ploidy level, basic chromosome number, and repetitive sequences content. For example, bread wheat (*Triticum aestivum* L.; 17 Gb; 2n = 6x = 42 chromosomes) is an allopolyploid, composed of six sets of chromosomes belonging to A, B, and D genomes with high content of non-coding, repetitive DNA. Contrastingly, *Brachypodium distachyon* has a small genome (272 Mb; 2n = 2x = 10 chromosomes) and low amount of repetitive sequences (Vogel and Hill 2008; International Brachypodium Initiative (IBI) 2010), making it a good model for structural genomic studies of grasses. It was previously reported that *B. distachyon* is a comparative and functional genetic model and its potential relevance for research on cereals, on the basis of its physiological and genetic advantages (Draper et al. 2001; Vogel and Bragg 2009). For example, the high degree of genetic synteny between wheat, *B. sylvaticum*, and *B. distachyon* enabled the identification of the genes present in the *Ph1* (**P**airing **h**omeologous 1) locus of wheat (Griffiths et al. 2006; Huo et al. 2009), demonstrating the potential application of the genus *Brachypodium* in comparative genomics. The phylogenetic relationships between *B. distachyon* and other cereals and grasses have been derived on the basis of the analysis of the internal transcribed spacer (ITS) and rDNA coding sequences (Hsiao et al. 1994), genomic markers, such as random amplified polymorphic DNA (RAPD), restriction fragment length polymorphism (RFLP) (Catalán et al. 1995), chloroplast restriction sites, and *ndh*F gene sequence (Kellogg 2001; Mochida and Shinozaki 2013). Those approaches showed that *B. distachyon* can be considered as a kind of missing link between rice (*Oryza sativa* L.) and temperate grasses.

Exploring the genomes of a wide range of plant species revealed the existence of highly conserved sequences and synteny in gene order. Comparative genomics can distinguish synteny, which means the presence of two or more loci on the same chromosome, and collinearity, which refers to the similarities in the physical order of loci (McCouch 2001). However, recently, the synteny is defined as the conservation of blocks of order within the sets of chromosomes which are being compared to each other. The first study of chromosome evolution and comparative genomics was performed between potato (*Solanum tuberosum*) and tomato (*Lycopersicon esculentum*) (Bonierbale et al. 1988). Initially, low copy RFLP markers and low resolution genetic maps were used to determine the phylogenetic relationships between Poaceae species, and established that most of the grass genomes are composed from 30 rice-independent linkage blocks (Gale and Devos 1998; Keller and Feuillet 2000). These methods enabled the macro-collinearity of grass genomes to be determined; however, they also overestimated the level of synteny. Recently, the development of additional genomic resources such as EST marker databases and whole genome sequences, combined with improved software, has enabled comparative genomic studies to reach a higher level of resolution, uncovering the micro-collinearity of sequences, which form the basis to establish a model of grass genome evolution (Devos 2005; Wei et al. 2007; Salse et al. 2008; Murat et al. 2014).

One of the main factors leading to chromosome evolution and speciation are changes in the amount and distribution of repetitive DNA sequences (Flavell et al. 1979; Cuadrado and Jouve 2002). It is hypothesized that these sequences play an important role in chromosome organization, stabilization of chromosome structure, recognition and segregation of chromosomes in mitosis and meiosis, and regulation of gene activity (Vershinin et al. 1995). Repetitive sequences account for up to 90 % of plant genomes (Heslop-Harrison 2000), and show diverse compositions in different genomes. Changes in repetitive sequences could be particularly useful in studies of genomic evolution. Some families of those sequences, e.g., *Afa* family (Nagaki et al. 1995), are present in more than one genus, which could be an evidence of their genomic relationship. Genomic in situ hybridization (GISH) can distinguish particular genomes at the chromosomal level. GISH can result in chromosome painting and show similarities of repetitive DNA distribution between related species. Moreover, the physical locations of conserved sequences can be visualized on chromosomes as well.

In this work, we compare both the model species (*Brachypodium distachyon*) as well as other important grasses, using genomic DNA probes. Our intention was to verify and classify cytologically visible similarities and differences in repetitive non-coding DNA sequences locations in given Pooideae species. A comparative approach using GISH, instead of comparison of selected sequences or specific chromosome regions, was performed to widen the understanding of the relationships within the Pooideae subfamily at the chromosomal level.

## Materials and methods

### Plant material

Ten species (Table 1) were selected for comparative GISH analysis: *Aegilops speltoides* Tausch., *Aegilops tauschii* Coss., *Brachypodium distachyon* L., *Festuca pratensis* L., *Hordeum vulgare* L., *Lolium perenne* L., *Secale cereale* L., *Triticosecale* Wittm., *Triticum aestivum* L., and *Triticum urartu* Tumanian ex Gandilyan. *Brachypodium distachyon* material was sourced from the University of Silesia in Katowice, Poland. Seeds of *Ae. speltoides*, *S. cereale*, *T. aestivum*, and *T. urartu* were kindly supplied for the study from the National Small Grains Germplasm Research Facility, National Small Grains Collection (Aberdeen, Idaho, USA). The remaining species were provided by a collection of the Institute of Plant Genetics, Polish Academy of Sciences in Poznan, Poland.

**Table 1:** Origin, chromosome number, size, and constitution of genomes of the studied Pooideae species according to Bennett and Leitch (2012)

No.	Species	Origin	Chromosome number (2n)	Genome size (1C; pg)	Genome constitution
1	*Aegilops speltoides* Tausch.	USDA (PI 542264)	14	5.15	SS
2	*Aegilops tauschii* Coss.	IPG PAS (D51)	14	5.1	DD
3	*Brachypodium distachyon* L.	US (Bd21)	10	0.36	BdBd
4	*Festuca pratensis* Huds.	IPG PAS (cv. ‘Skra’)	14	2.23	FpFp
5	*Hordeum vulgare* L.	IPG PAS (cv. ‘Georgia’)	14	5.55	HH
6	*Lolium perenne* L.	IPG PAS (cv. ‘Arka’)	14	2.76	LpLp
7	*Secale cereale* L.	USDA (PI 323382; cv. ‘Imperial’)	14	8.28	RR
8	*Triticum aestivum* L.	USDA (Cltr 14108; cv. ’Chinese Spring’ )	42	17.33	AABBDD
9	*Triticum urartu* Tumanian ex Gandilyan	USDA (PI 428335)	14	4.93	AA
10	×*Triticosecale* Wittm.	IPG PAS (cv. ‘Kitaro’)	42	19.80	AABBRR

### Chromosome preparation

Seeds of each species were germinated on filter paper in Petri dishes for 3–4 days in the dark. The root tips were immersed in ice-cooled water for 26 h, fixed in ethanol and acetic acid (3:1, v/v), and stored at −20 °C until required. Mitotic chromosome preparations were made from root tips digested in a mixture of enzymes, diluted with 0.01 M sodium citric buffer, containing 20 % (v/v) pectinase (Sigma), 1 % (w/v) cellulose (Calbiochem), and 1 % (w/v) cellulase ‘Onozuka R-10’ (Serva). Meristems were dissected from root tips, squashed in drops of 45 % acetic acid, and the good quality preparation was frozen (Hasterok et al. 2006).

### DNA isolation and probe labeling

Genomic DNA (gDNA) from young leaves of all selected species were isolated with the C-TAB method (Doyle and Doyle 1990). DNA from all plants were obtained at the same stage. After the extraction of DNA, samples were labeled by nick translation with tetramethylrhodamine-5-dUTP (Sigma-Aldrich). The ribosomal sequences 35S rDNA and 5S rDNA were labeled with digoxigenin-11-dUTP by nick translation and with tetramethyl-rhodamine-5-dUTP (Sigma-Aldrich) by polymerase chain reaction (PCR), respectively (Kwiatek et al. 2016b).

### In situ hybridization

The GISH procedures were performed according to the protocol of Kosmala et al. (2006) and Kwiatek et al. (2016a, b), with minor modifications. The GISH protocol was standardized by several repeats to ensure that the obtained results were comparable and reproducible. The hybridization mixture consisted of 50 % deionized formamide, 10 % dextran sulfate, 2 × SSC, 0.5 % SDS, as well as 100–120 ng/slide gDNA probe. The blocking DNA was not used. Several initial GISH experiments were carried out to reveal optimal specifications, such as probe concentrations and wash temperatures. After establishing optimal GISH conditions, we used only those conditions for all experiments described in this paper. Chromosome preparations and hybridization mixture were denatured together at 80 °C for 2 min and then hybridized overnight at 37 °C. The post-hybridization washes were performed, according to Heslop-Harrison (2000), at 42 °C in 2 × SSC buffer. Probes labeled with tetramethylrhodamine-5-dUTP were directly visualized. After the acquisition of images, selected slides were washed off and reprobed with a new set of probes (35S rDNA and 5S rDNA). The reprobing procedures were performed according to the protocol of Heslop-Harrison et al. (1992), with minor modifications. Immunodetection of the digoxigenated probe (35S rDNA) was performed using fluorescein isothiocyanate-conjugated anti-digoxigenin antibody (Sigma-Aldrich). Mitotic cells were examined with an Olympus XM10 CCD camera attached to an Olympus BX61 automatic epifluorescence microscope. Image processing was carried out using Olympus Cell-F imaging software and Micrografx Picture Publisher software.

## Results

GISH analyses were performed in order to determine the relationships between total genomic DNA of key crops and forage species at the chromosomal level. The experiments were carried out using labeled probes from each species to hybridize with chromosomes of every species used in this study based on a “round robin” rule. Overall, 100 GISH experiments were used (10 probes × 10 species, Table 2). The experiments showed various types of signal locations, including: telomeric and centromeric regions, rDNA loci, subcentromeric and subtelomeric regions, as well as chromosome labeling or signals dispersed along whole chromosomes. Additionally, rDNA-FISH (ribosomal DNA fluorescent in situ hybridization) was carried out when GISH revealed rDNA-like signals.

**Table 2:** Specification of comparative mapping of the Pooideae subfamily according to a “round robin” rule

Species	*B. distachyon*	*H. vulgare*	*S. cereale*	*T. aestivum*	*Ae. tauschii*	*Ae. speltoides*	*T. urartu*	×*Triticosecale*	*F. pratensis*	*L. perenne*
No. of rDNA loci	35S 2 loci; 5S 2 loci	35S 4 loci; 5S 8 loci	35S 2 loci; 5S 4 loci	35S 4 loci; 5S 12 loci	35S 2 loci; 5S 4 loci	35S 4 loci; 5S 2 loci	35S 4 loci; 5S 4 loci	35S 6 loci; 5S 10 loci	35S 2 loci; 5S 2 loci	35S 7 loci; 5S 2 loci
*B. distachyon*	Centromeric regions + 2 signals of 35S rDNA	4 signals of 35S rDNA (NOR chromosomes 6 and 7)	Telomeric regions + 2 signals of 35S + 4 signals of 5S rDNA	Telomeric regions + 4 signals of 35S rDNA	Telomeric regions + 2 signals of 35S rDNA	4 signals of 35S + 2 signals of 5S rDNA, centromeric regions	Telomeric regions + 4 signals of 35S rDNA	Telomeric regions + 6 signals of 35S + 10 signals of 5S rDNA	Telomeric regions + 2 signals of 35S rDNA	Telomeric regions + 7 signals of 35S rDNA
*H. vulgare*	35S rDNA	Chromosome labeling + centromeric regions	Chromosome labeling + 2 pericentromeric signals in chromosomes without rDNA loci	Chromosome labeling, in some chromosomes lack of signals in centromeric and terminal regions	Labeling of chromosome segments	Chromosome labeling + pericentromeric signals	Chromosome labeling + 2 pericentromeric signals	Pericentromeric signals in 14 chromosomes of B genome	Chromosome labeling + 2 signals of 35S rDNA	Chromosome labeling + 7 signals of 35S rDNA
*S. cereale*	Strong dispersed signals + 2 signals of 35S + 2 signals of 5S rDNA	Centromeric regions	Chromosome labeling + telomeric regions	Chromosome labeling and signals in chromosomes without rDNA loci	Chromosome labeling + 6 terminal signals in chromosomes without rDNA loci	Chromosome labeling without terminal regions, 4–5 signals in chromosomes without rDNA loci	Chromosome labeling	Labeling of 14 chromosomes (genome R)	Weak chromosome labeling + 2 signals of 35S rDNA	Strong, dispersed signals
*T. aestivum*	35S rDNA + 5S rDNA + telomeric regions + centromeric regions	Pericentromeric regions	Very weak chromosome labeling without NORs and telomeric regions	Chromosome labeling	Chromosome labeling	Chromosome labeling without terminal regions + pericentromeric signals	Chromosome labeling without terminal regions (1 arm in 2 chromosomes)	Labeling of 28 chromosomes (genomes A and B), very weak labeling of 14 R-genome chromosomes	Chromosome labeling + 2 signals of 35S rDNA	7 signals of 35S rDNA
*Ae. tauschii*	35S rDNA + telomeric regions + centromeric regions	Chromosome labeling	Chromosome labeling without telomeric regions	Chromosome labeling	Chromosome labeling + telomeric regions + signals in chromosomes without rDNA loci	Chromosome labeling without terminal regions	Chromosome labeling (8 chromosomes with stronger intensity)	Chromosome labeling, 14 chromosomes with stronger intensity (genome B)	Weak chromosome labeling + 2 signals of 35S rDNA	Chromosome labeling + 5 signals of 35S rDNA
*Ae. speltoides*	2 signals of 35S + 2 signals of 5S rDNA + centromeric regions	Pericentromeric regions + centromeric regions	Chromosome labeling without terminal regions	14 signals of 5S rDNA, centromeric regions in 14 chromosomes of B genome	Chromosome labeling + 2 terminal signals in 1 pair without rDNA loci	Chromosome labeling + pericentromeric regions	Chromosome labeling without terminal regions in 1 pair	Labeling of 14 chromosomes (genome B)	Chromosome labeling + 2 signals of 35S rDNA	Weak chromosome labeling + 7 signals of 35S rDNA
*T. urartu*	2 signals of 35S rDNA	Chromosome labeling without terminal regions	Chromosome labeling without terminal regions	Chromosome labeling, 14 chromosomes with stronger intensity (genome A)	Chromosome labeling without terminal regions	Chromosome labeling without terminal regions	Chromosome labeling	Chromosome labeling (A genome - strong signals, B - weaker intensity, R - very weak labeling)	Weak chromosome labeling + 2 signals of 35S rDNA	Weak chromosome labeling + 7 signals of 35S rDNA
×*Triticosecale*	35S rDNA + 5S rDNA + strong dispersed signals	Pericentromeric regions	Chromosome labeling without 2 NORs + telomeric regions	Chromosome labeling without terminal regions, centromeric regions and 2 NORs	Chromosome labeling without terminal regions and NORs	Chromosome labeling of selected regions + pericentromeric signals	Chromosome labeling (6 chromosomes with stronger intensity; 2 chromosomes without labeling of terminal regions)	Chromosome labeling	2 signals of 35S rDNA	Chromosome labeling
*F. pratensis*	35S rDNA + centromeric regions	Chromosome labeling + centromeric regions	Chromosome labeling without terminal regions	Weak dispersed signals	Dispersed signals + telomeric regions (not in all chromosomes)	Chromosome labeling without terminal regions and interstitial block in chromosome 5	Chromosome labeling	Chromosome labeling	Chromosome labeling	Chromosome labeling
*L. perenne*	2 signals of 35S + 2 signals of 5S rDNA + telomeric regions	Chromosome labeling + 4 signals of 35S rDNA	Chromosome labeling without terminal regions + 2 signals of 35S rDNA	4 signals of 35S rDNA + telomeric regions	2 signals of 35S + 2 signals of 5S rDNA + telomeric regions + weak dispersed signals	4 signals of 35S + 2 signals of 5S rDNA	4 signals of 35S rDNA + terminal signals in 2 chromosomes + dispersed signals	Chromosome labeling + 6 signals of 35S rDNA	Strong, distinct signals	Chromosome labeling without terminal regions in 1 pair

Columns = chromosome preparations; *rows* = gDNA probes

### *Brachypodium distachyon* chromosomes

**Fig. 1:**
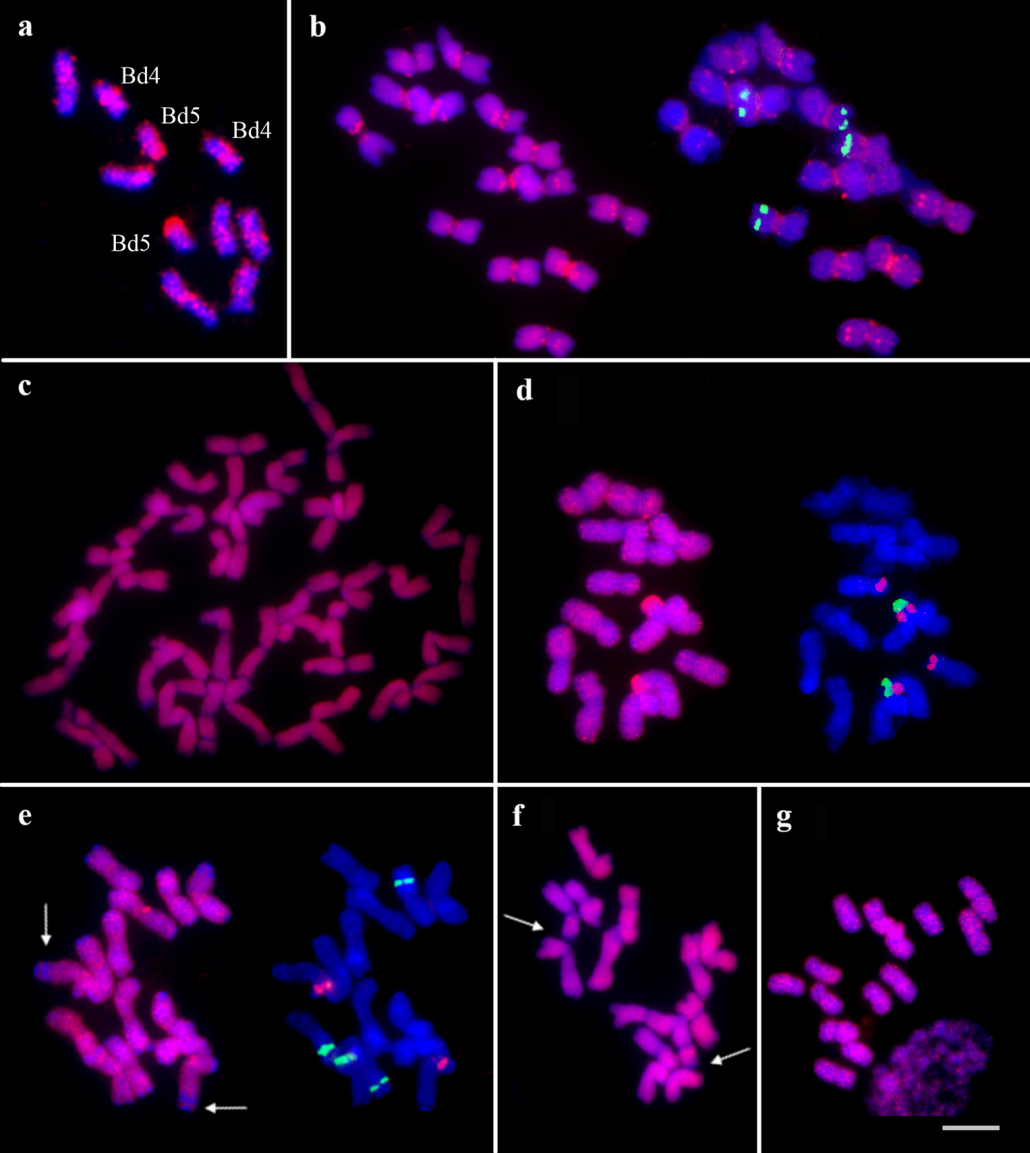
Comparative GISH mapping within the Pooideae subfamily using gDNA (*red*) of selected species: **a** *Ae. speltoides* gDNA probe to *B. distachyon* chromosomes; **b** *Ae. speltoides* gDNA probe to *H. vulgare* chromosomes; **c** *Ae. tauschii* gDNA probe to *T. aestivum* chromosomes; **d** *L. perenne* gDNA probe to *Ae. tauschii* chromosomes; **e** *F. pratensis* gDNA probe to *Ae. speltoides* chromosomes; **f** *Ae. speltoides* gDNA probe to *T. urartu* chromosomes; **g** ×*Triticosecale* gDNA probe to *L. perenne* chromosomes. **b**, **d**, and **e**: on the right site are shown metaphase plates with 35S (*green*) and 5S rDNA (*red*). The *white arrows* indicate unlabeled regions in chromosomes. Scale bar = 5 μm

The GISH experiments on *B. distachyon* (2n = 2x = 10) chromosomes resulted in the 35S rDNA loci being identified using all probes of gDNA (genomic DNA) probes tested. The 5S rDNA loci were labeled by *S. cereale*, *T. aestivum*, *Ae. speltoides* (Fig. 1a), ×*Triticosecale*, and *L. perenne* gDNA probes only. Pericentromeric signals were observed when *B. distachyon*, *T. aestivum*, *Ae. tauschii*, *Ae. speltoides* (Fig. 1a), and *F. pratensis* gDNA probes were used. Rye and triticale probes also gave strong and clear signals dispersed in all *B. distachyon* chromosomes. Furthermore, *B. distachyon* telomeric regions were labeled when *T. aestivum*, *Ae. tauschii*, and *L. perenne* DNA were used as genomic probes.

### *Hordeum vulgare* chromosomes

**Fig. 2:**
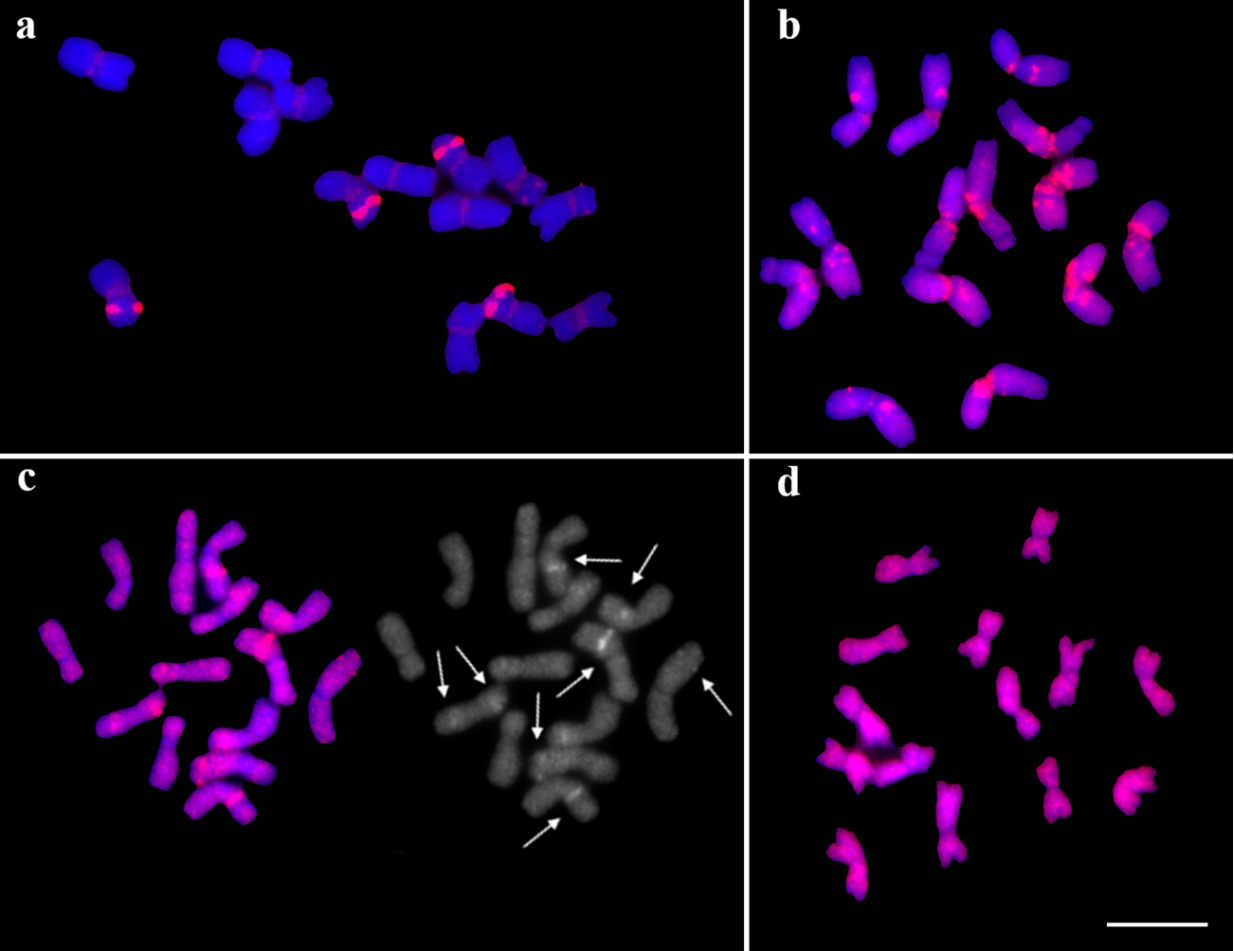
Hybridization patterns for comparative mapping of gDNA (*red*): **a** *B. distachyon* gDNA probe to *Ae. speltoides* chromosomes showing centromeric regions, 5S and 35S rDNA; **b** ×*Triticosecale* gDNA probe to *H. vulgare* chromosomes: pericentromeric and peritelomeric signals; **c** *L. perenne* gDNA probe to *F. pratensis* chromosomes: other distinct, strong signals (*white arrows*); **d** *S. cereale* gDNA probe to *T. urartu* chromosomes: chromosome labeling and dispersed signals. Scale bar = 5 μm

All barley (*H. vulgare*; 2n = 2x = 14) chromosomes were labeled by *H. vulgare*, *Ae. tauschii*, *F. pratensis*, and *L. perenne* gDNA probes. By contrast, *T. urartu* probe marked every barley chromosome but left the telomeric regions unlabeled. Clear signals in the centromeric regions were observed when *H. vulgare*, *S. cereale*, *Ae. speltoides* (Fig. 1b), and *F. pratensis* gDNA probes were hybridized. In addition, pericentromeric regions were identified when *T. aestivum*, *Ae. speltoides*, and ×*Triticosecale* (Fig. 2b) gDNA probes were used. Hybridization using gDNA probes generated from *B. distachyon* and *L. perenne* resulted in four strong signals, which corresponded to the 35S rDNA loci.

### *Secale cereale* chromosomes

**Fig. 3:**
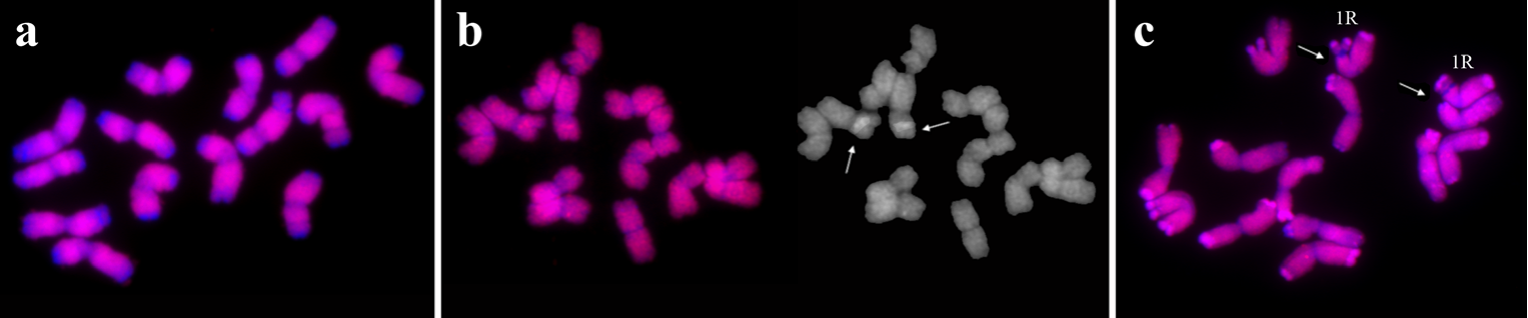
Labeling of all chromosomes using gDNA (*red*) of selected species beyond: **a** subtelomeric regions: *T. urartu* gDNA probe to *Ae. speltoides* chromosomes; **b** centromeric regions: *H. vulgare* gDNA probe to *T. urartu* chromosomes (the *arrows* indicate distinct pericentromeric signals); **c** NORs: triticale gDNA probe to *S. cereale* chromosomes (the *arrows* indicate NORs)

All rye (*S. cereale*; 2n = 2x = 14) chromosomes were labeled by *H. vulgare*, *S. cereale*, and ×*Triticosecale* probes. The triticale probe did not hybridize with two 35S rDNA loci (Fig. 3c). The *H. vulgare* probe also identified an additional two signals located on the pericentromeric regions of the chromosomes lacking rDNA loci. Hybridizations with the rest of probes, except for *B. distachyon* probe, resulted in labeling of all the rye chromosome except for the telomeric regions. Similarly to triticale, the wheat probe also did not label two 35S rDNA loci on rye chromosomes. In contrast, *B. distachyon* gDNA probe gave signals in two 35S and four 5S rDNA loci, as well as in terminal regions. Finally, the *L. perenne* probe highlighted an additional two regions which corresponded to the 35S rDNA loci.

### *Triticum aestivum* chromosomes

All wheat chromosomes (*T. aestivum*; 2n = 6x = 42) were labeled by *T. aestivum*, *T. urartu*, *Ae. tauschii* (Fig. 1c), and *S. cereale* gDNA probes, although the *T. urartu* probe resulted in 14 chromosomes of A genome being marked stronger. The rye gDNA probe showed strong and dispersed signals in most of the wheat chromosomes. Triticale and barley gDNA probes also labeled all chromosomes; however, the centromeric and telomeric regions as well as two 35S rDNA loci (only in the case of triticale probe) remained unlabeled. Contrastingly, the *Ae. speltoides* gDNA probe gave only 14 signals in 5S rDNA loci and 14 centromeric regions in B genome chromosomes. The *B. distachyon* probe resulted in labeled telomeric sites and four signals in 35S rDNA loci, while *L. perenne* yielded four 35S rDNA loci and telomeric regions. Finally, weak and dispersed signals were observed using *F. pratensis* gDNA probe.

### *Aegilops tauschii* chromosomes

*Aegilops tauschii* (2n = 2x = 14) chromosomes were marked in GISH experiments using *T. aestivum*, *Ae. tauschii*, *S. cereale*, and *Ae. speltoides* gDNA probes. Additionally, *Ae. tauschii* probe hybridization resulted in strong telomeric signals and clear dispersed signals along all chromosomes. Six telomeric sites were detected using rye probe. The *Ae. speltoides* gDNA probe gave also two terminal signals in *Ae. tauschii* chromosomes. The ×*Triticosecale* and *T. urartu* gDNA probes also marked all chromosomes; however, the telomeric regions and two 35S rDNA loci (only in the case of triticale probe) remained unlabeled. Labeled segments of *Ae. tauschii* were observed when *H. vulgare* gDNA probe was used in the GISH experiments. In contrast, *L. perenne* (Fig. 1d) and *F. pratensis* gDNA probes resulted in weak dispersed sites observed in all chromosomes. Additionally, the *L. perenne* probe gave signals in two 35S and two 5S rDNA loci, and also labeled all telomeric regions (Fig. 1d). Two 35S rDNA loci and telomeric sites were also labeled when GISH was carried out using *B. distachyon* gDNA probe.

### *Aegilops speltoides* chromosomes

The experiments with labeled DNA of *Ae. tauschii*, *T. urartu* (Fig. 3a), *S. cereale*, *T. aestivum*, and *F. pratensis* (Fig. 1e) revealed labeling of all *Ae. speltoides* (2n = 2x = 14) chromosomes with the exception of the telomeric regions, whereas gDNA probe of *S. cereale* revealed strong, distinct signals on several chromosomes. GISH with *Ae. speltoides* gDNA probe resulted in chromosome labeling and brighter signals in pericentromeric regions. Similar results for all chromosomes of *Ae. speltoides* were obtained when gDNA of ×*Triticosecale*, *T. aestivum*, and *H. vulgare* were used for GISH. What is more, the subtelomeric region of a pair of chromosomes possessing the 5S loci remained unlabeled when hybridization with *F. pratensis* gDNA (Fig. 1e) was performed. Four 35S and two 5S rDNA loci were obtained for the DNA of *L. perenne* and *B. distachyon* (Fig. 2a) and centromeric regions were highlighted using *B. distachyon* only.

### *Triticum urartu* chromosomes

Labeled gDNA of five species, *S. cereale* (Fig. 2d), *Ae tauschii*, *T. urartu*, *F. pratensis*, and *H. vulgare* lead to chromosome labeling on their entire length. Additionally, distinct signals in the pericentromeric regions in two chromosomes and unlabeled centromeric regions in all chromosomes were observed in experiments using *H. vulgare* DNA (Fig. 3b). Chromosome painting with lack of signals in the terminal part of one pair of chromosomes was obtained for the DNA of *Ae. speltoides* (Fig. 1f), *T. aestivum*, and ×*Triticosecale*. Additionally, *Ae. speltoides* probe resulted in some strong, clear signals in pericentromeric regions (Fig. 1f). *Brachypodium distachyon* probe resulted in strong subtelomeric signals and gave four signals of 35S rDNA loci. The genomic DNA of *L. perenne* revealed only the 35S rDNA sequences.

### ×*Triticosecale* chromosomes

Hybridization with gDNA of *B. distachyon* and *H. vulgare* to chromosomes of triticale (×*Triticosecale*; 2n = 6x = 42) provided contrasting results to the remaining experiments, where chromosome labeling was observed. The gDNA of *B. distachyon* was mapped in telomeric sites and gave six signals of 35S rDNA loci and ten signals of 5S rDNA loci. The dispersed, strong signals located near the centromeric and telomeric regions in genome B were visible for the experiment with gDNA of *H. vulgare*. GISH with *S. cereale* probe enabled to distinguish the R genome chromosomes, while the DNA probe of *Ae. speltoides* visualized the B genome, and DNA of *T. urartu* the A genome chromosomes. What is more, the hybridization with DNA of *T. aestivum* enabled to identify A and B genome as well as R genome chromosomes in terms of negative discrimination (very weak labeling). Probing of homologous gDNA to triticale chromosomes resulted in entire chromosomes being labeled. Finally, probing with *Ae. tauschii* gDNA resulted in chromosome labeling, but with more intensity of the B genome of ×*Triticosecale*.

### *Festuca pratensis* chromosomes

GISH with gDNA probes of selected species, with the exception of *F. pratensis* and *L. perenne* (Fig. 2c), revealed two signals of 35S rDNA in the karyotype of *F. pratensis* (2n = 2x = 14). Furthermore, in nearly all cases where 35S rDNA signals were detected, complete chromosome labeling was also observed; the exceptions were *B. distachyon* and ×*Triticosecale*. However, *B. distachyon* probe produced weak signals in telomeric regions. Chromosome labeling was clearly visible when *F. pratensis* gDNA was used to probe chromosomes of *F. pratensis*. Hybridization of *L. perenne* DNA to *F. pratensis* revealed strong, distinct signals in some chromosomes (Fig. 2c).

### *Lolium perenne* chromosomes

The hybridization of genomic DNA of *B. distachyon*, *H. vulgare*, *T. aestivum*, *Ae. tauschii*, *Ae. speltoides*, and *T. urartu* to chromosomes of *L. perenne* (2n = 2x = 14) identified the 35S rDNA loci. What is more, the *B. distachyon* gDNA probe labeled the telomeric sites of *L. perenne*. Strong, dense signals were observed for *S. cereale* probe. *Lolium perenne* chromosomes probed with gDNA from *H. vulgare*, *Ae. tauschii*, *T. urartu*, and *Ae. speltoides* resulted in both 35S rDNA signals and chromosome labeling. Probing gDNA of ×*Triticosecale* (Fig. 1g), *F. pratensis*, and *L. perenne* resulted in the labeling of all chromosomes. GISH with gDNA of *L. perenne* to *L. perenne* chromosomes showed an unlabeled terminal part of a pair of chromosomes that lacked rDNA loci.

## Discussion

In this study, we detected and described the hybridization patterns of various genomic DNA probes generated from key representatives of cereals, forage grasses, and *B. distachyon* on mitotic chromosomes of those species. The aim of this approach was to score the affinity of the given species by using gDNA as probes, without blocking DNA from the species chosen for chromosome spreads preparations. As a result, the whole chromosomes or chromosome regions were distinguished or variable types of signals were visualized to prove the different levels of the relationships between genomes used in this study.

A part of the presented results are either as they were expected or confirm the results reported in other papers. However, our study also reveals some novel insights, which require more specific analysis in the future. The most characteristic DNA motifs in the Pooideae subfamily are telomeric and rDNA sequences. Our results confirmed the previous study of Hasterok et al. (2004), who reported labeled pericentromeric regions and 35S rDNA loci on *B. distachyon* chromosomes using *B. distachyon* genomic probe. This phenomenon could be explained by the small content of repetitive sequences in this genome. Besides, Mayer et al. (2011) compared the gene order of *B. distachyon* with *H. vulgare* and reported that the number of conserved syntenic loci was high (14,422), reflecting a closer phylogenetic affinity. What is more, Ma et al. (2010) carried out GISH reciprocal experiments between *B. distachyon* and *H. vulgare* and observed mainly 35S ribosomal DNA loci. In the present study, the same type of affinity was observed in GISH runs with all of the gDNA probes, indicating that this high copy DNA sequence is conserved between analyzed grasses. Ribosomal DNA loci, which are essential for all organisms, can be detected at the cytogenetic level; however, homogenization operates very strongly in these regions in all Poaceae genomes (Sallares and Brown 1999). Signals observed in the centromeric regions of *B. distachyon* led to the assumption that this species is more closely related to *Ae. speltoides* and *Ae. tauschii* than with *T. urartu*. Moreover, we showed a certain degree of genomic relationship between *B. distachyon* and *F. pratensis*. This point of view could also be confirmed by the phylogenetic analysis of grass species based on the *ndf*H chloroplast gene sequence made by Mochida and Shinozaki (2013).

Considering their importance in agriculture, the most analyzed temperate cereal species in comparative genomics are barley and wheat, along with their relatives. Based on comparison to the previously constructed high-density physical marker map of wheat (Qi et al. 2004), Mayer et al. (2011) reported that barley contains an archetypal Triticeae genome. Most of the chromosome arms showed well-conserved synteny with previously reported chromosomal translocations (Qi et al. 2006). In the present work, we observed labeled pericentromeric regions on *H. vulgare* chromosomes using *Ae. speltoides*, *T. aestivum*, and ×*Triticosecale* gDNA probes. These observations are correlated with the results of Icsó et al. (2015), where gDNA of *H. vulgare* was used for the chromosome probing of *T. aestivum*. They reported that gDNA of barley produced hybridization bands mainly in the pericentromeric and intercalary chromosome regions of B genome chromosomes and it corresponded to the GAA FISH pattern. Surprisingly, we observed differences in the labeling of wheat chromosomes using barley genomic probe in comparison to chromosomes of wheat labeled with gDNA probes of its ancestral species. The three genomes of wheat share similar repetitive sequence types, with the D genome specific repetitive sequences being the most frequent (Nagaki et al. 1995; Jia et al. 2013). In addition, the existence of tandemly repeated sequence dpTa1 presented in 58 species of the Triticeae tribe have been reported (Vershinin et al. 1994). The occurrence of signals detected in phylogenetically distant species may indicate strong relationships between repetitive sequences in selected grasses. For example, the pSc119.2 sequence from rye is widely used in the identification of wheat chromosomes and its relatives (Cuadrado and Jouve 1994; Schneider et al. 2003, 2005; Wiśniewska et al. 2013).

On the other hand, we have observed an unexpected lack of signals in secondary constrictions of rye chromosomes probed by triticale genomic DNA. This may be connected with the differences in rDNA sequences between 1R chromosomes of rye and 1R chromosomes of triticale. In general, unlabeled chromosome regions can be related with species-specific sequences or disparate pathways of chromosome differentiation, which was exposed by the lack of labeled segments of *Ae. speltoides* chromosomes probed with *F. pratensis* gDNA. The most likely explanation for the unlabeled telomeric regions could be the fact that the *Ae. speltoides* genome carries subtelomeric sequences Spelt1 and Spelt52 (Salina et al. 2006, 2009). On the other hand, the lack of signals from the 5S chromosome segment of *Ae. speltoides* remains unknown but indicates that this region could have gone under structural changes during S genome evolution. What is more, we observed single signals in pericentromeric locations in some chromosomes of *T. urartu* probed with *Ae. speltoides* gDNA. Similarly, Belyayev et al. (2000) reported pericentromeric signals in the chromosomes of *T. urartu* and suggested that those regions are conserved in A and B genomes. Moreover, comparative GISH resulted in a lack of signals in the subtelomeric regions in the chromosomes of *T. aestivum*, *Ae. speltoides*, *Ae. tauschii*, and *H. vulgare*. This could be determined by the different constitution of subtelomeric regions. For example, the composition of the termini of *S. cereale* chromosomes is complex and, besides the basic telomeric sequence (T/A)_1-4_ G_1-8_, it also contains tandemly organized DNA families such as pSc119.2, pSc200, and pSc250 (Salina et al. 2009).

Our comparative analysis of genomic relationships within crucial Pooideae species revealed some intriguing results, such as unexpected chromosome labeling between distant species (e.g., ×*Triticosecale* vs. *L. perenne*), dispersed signals (e.g., *L. perenne* vs. *F. pratensis*), or labeling of selective chromosome regions (e.g., *S. cereale* vs. *Ae. speltoides*). The strong, distinct signals observed for *L. perenne* and *F. pratensis* can be useful for the karyotyping of *F. pratensis* chromosomes. Till now, only two pairs of chromosomes, 2F with 35S rDNA and 3F with 5S rDNA loci, can be easily identifiable (Jones et al. 2002; Thomas 1981).

In conclusion, our results are in parallel with the comparative studies made on model species and key representatives of the Pooideae subfamily. However, some results indicated unknown intergenomic dependencies that need further analysis. Moreover, reciprocal GISH analysis on *Aegilops*, *Hordeum*, and *Triticum* species could shed light on the chromosome structure and evolution within the Triticeae tribe.
